# Editorial: State of the art in acute care surgery: application, innovation, and future perspectives

**DOI:** 10.3389/fsurg.2026.1961242

**Published:** 2026-08-31

**Authors:** Nikolaos Pararas

**Affiliations:** Third Department of Surgery, National and Kapodistrian University of Athens, Attikon University Hospital, Athens, Greece

**Keywords:** acute care surgery, clinical decision-making, future perspectives, innovations, minimally invasive surgery

**Editorial on the Research Topic**
State of the art in acute care surgery: application, innovation, and future perspectives

## Introduction

Acute care surgery (ACS) has undergone a remarkable transformation over the past two decades. What initially emerged as a practical response to fragmented emergency surgical services has developed into a comprehensive specialty encompassing emergency general surgery, trauma, surgical critical care, rescue surgery, and the management of complex postoperative complications. The modern acute care surgeon is expected to move seamlessly between rapid diagnosis, operative decision-making, intensive care, and multidisciplinary coordination, often while managing patients whose physiological condition evolves by the hour. In this setting, technical expertise alone is no longer sufficient; successful care depends equally on clinical judgement, adaptability, and the ability to integrate evolving evidence into time-critical decisions.

The contributions gathered in this Research Topic capture the breadth of that evolution. They span the spectrum of contemporary ACS, from common emergencies such as appendicitis, cholecystitis, bowel obstruction, gastrointestinal perforation, and pancreatitis to rare anatomical variants, unusual traumatic injuries, complex wound management, and organizational challenges within emergency surgical systems. Although the individual studies differ considerably in methodology and clinical focus, they collectively reflect the increasingly multidisciplinary nature of emergency surgical care.

Several recurring themes emerge throughout the Research Topic. Advances in diagnostic imaging, predictive modelling, and risk stratification are enabling more individualized decision-making during the earliest stages of patient assessment. At the same time, minimally invasive surgery, therapeutic endoscopy, and image-guided interventions continue to expand the range of emergency conditions that can be managed without conventional open surgery. These technical developments are accompanied by equally important progress in perioperative care, including enhanced recovery protocols, antimicrobial stewardship, structured critical care, abdominal wall reconstruction, and specialized nursing practice. Together, these advances demonstrate that improvements in ACS increasingly arise from coordinated systems of care rather than from isolated procedural innovation.

Perhaps the most striking observation across the Research Topic is the emphasis on continuity of care. Emergency surgery no longer begins and ends in the operating theatre. Decisions made during the initial assessment influence operative planning, postoperative recovery, rehabilitation, and ultimately long-term functional outcomes. Likewise, advances in trauma systems, digital decision-support platforms, and multidisciplinary collaboration illustrate that organizational innovation has become as important as technical refinement in determining patient outcomes.

The studies presented here therefore represent more than a collection of individual clinical experiences. Viewed together, they provide a contemporary perspective on how ACS continues to evolve as a specialty that combines technical excellence with evidence-based decision-making, multidisciplinary collaboration, and patient-centred care. The following sections examine these contributions within a thematic framework, illustrating how they collectively define current practice while identifying the priorities that are likely to shape the future of acute care surgery.

### Precision diagnosis and decision-making in emergency general surgery

Accurate diagnosis remains the foundation of acute care surgery, yet contemporary practice increasingly demands more than identifying the underlying pathology. Surgeons must determine not only who requires immediate operation but also who may benefit from staged intervention, non-operative treatment, or minimally invasive alternatives. Several studies in this collection demonstrate how improved risk stratification, advanced imaging, and structured clinical assessment are refining these decisions while reinforcing the central importance of clinical judgement.

This evolution is well illustrated by Lin et al., who review contemporary approaches to predicting intestinal ischemia in strangulated small bowel obstruction. Rather than relying on isolated laboratory values or radiological signs, they describe a shift toward integrated models that combine serial clinical assessment, biochemical markers, imaging findings, and emerging computational tools. Their review also provides an important reminder that increasingly sophisticated prediction models have value only if they remain interpretable, externally validated, and practical in time-critical emergency settings.

The interpretation of imaging findings presents a similar challenge. Ji et al. show that hepatic portal venous gas should be viewed as a radiological sign rather than a diagnosis, with clinical significance determined by the underlying pathology. Cross-sectional imaging defines the anatomical problem, but the decision to operate still depends on the patient's physiological condition, abdominal examination, and the likelihood of bowel ischemia. Imaging therefore refines clinical judgement rather than replacing it.

Rare presentations continue to test diagnostic reasoning. Wang et al. report adult intestinal malrotation presenting as small bowel obstruction, whereas Zhuang describes omental infarction during pregnancy initially mistaken for acute appendicitis. Although both conditions are uncommon, they illustrate the importance of maintaining a broad differential diagnosis when conventional explanations fail. In these situations, diagnostic laparoscopy serves both as a definitive diagnostic tool and, when appropriate, as the initial therapeutic intervention.

Two studies broaden the discussion by examining how healthcare organization influences disease severity. During the COVID-19 pandemic, Obeidat et al. observed fewer admissions for acute appendicitis but a greater proportion of perforated cases requiring open surgery, suggesting that delayed presentation altered the natural history of disease. Fu et al. similarly demonstrated marked regional differences in the recovery of elective surgical services, highlighting how healthcare capacity and organizational resilience directly influence emergency surgical outcomes.

Finally, Zhang et al. extend precision medicine beyond operative decision-making by developing a validated model predicting moderate-to-severe pain after laparoscopic appendectomy. Anticipating postoperative pain allows earlier implementation of individualized analgesic strategies and more realistic patient counselling, reinforcing the growing emphasis on patient-centred recovery alongside traditional surgical outcomes.

Taken together, these studies illustrate that precision in acute care surgery increasingly arises from integrating clinical examination, imaging, prediction models, and healthcare context into a single decision-making process. Technology enhances this process, but experienced surgical judgement remains its essential component.

### Innovation in source control, minimally invasive surgery, and perioperative recovery

The expanding role of minimally invasive surgery is one of the clearest indicators of how acute care surgery has evolved. Across the studies included in this Research Topic, laparoscopy and therapeutic endoscopy are presented not as alternatives to established surgical principles but as complementary approaches that broaden treatment options when patient physiology, anatomy, and local expertise are appropriately aligned.

This concept is illustrated by Jiang et al., who successfully performed laparoscopic cholecystectomy with common bile duct exploration in a patient with **situs inversus totalis**. Although mirror-image anatomy demands considerable technical adaptation, careful preoperative planning and modified operative strategy allowed definitive treatment without sacrificing the benefits of minimally invasive surgery. Similarly, Wang et al. report fluorescence-guided laparoscopic central pancreatectomy for traumatic pancreatic transection, demonstrating that organ-preserving surgery may be feasible in carefully selected, hemodynamically stable patients. A comparable philosophy underlies the laparoscopic management of penetrating liver trauma described by Zhang et al., where meticulous imaging assessment and controlled operative technique permitted definitive treatment while avoiding unnecessary laparotomy. Collectively, these reports reinforce that minimally invasive trauma surgery should be reserved for appropriately selected patients rather than viewed as a universal replacement for conventional operative approaches.

Therapeutic endoscopy has followed a similar trajectory. Zhou et al. successfully managed perforation of a duodenal diverticulum caused by an impacted chicken bone through endoscopic foreign-body extraction and defect closure, while Zhang and Zhang demonstrated that selected perforations occurring during endoscopic ultrasound can be treated effectively with over-the-scope clips. In both situations, prompt diagnosis, careful evaluation of contamination, and immediate access to advanced endoscopic expertise were critical determinants of success. These experiences highlight how endoscopy has become an integral component of emergency source control rather than simply a diagnostic adjunct.

The collection also emphasizes that innovation extends beyond operative technique to encompass perioperative management. Wang et al., through a systematic review and meta-analysis, support the adaptation of enhanced recovery principles to patients undergoing surgery for gastrointestinal perforation. Although emergency surgery differs fundamentally from elective practice, interventions such as optimized fluid therapy, multimodal analgesia, early mobilization, and timely nutritional support appear capable of improving recovery when tailored to the patient's physiological condition.

Individualized perioperative decision-making is further illustrated by acute biliary disease. Lluís et al. demonstrate that index-admission cholecystectomy frequently provides better long-term outcomes than conservative management or percutaneous drainage in selected elderly patients, supporting treatment decisions based on physiological reserve rather than chronological age alone. Complementing this work, Váczi et al. characterize bile microbiology in acute cholecystitis and highlight the growing importance of culture-guided antimicrobial therapy in the era of multidrug-resistant organisms.

Finally, Subasinghe et al. examine the management of necrotizing pancreatitis within a tertiary hepatopancreatobiliary centre, showing how contemporary step-up strategies must be adapted to local resources while preserving the principles of delayed intervention, effective sepsis control, and multidisciplinary care.

Across these studies, the common message is clear: technological progress improves emergency surgical care only when integrated with careful patient selection, sound operative judgement, and coordinated perioperative management. Innovation therefore lies not simply in performing less invasive procedures, but in choosing the intervention most likely to achieve definitive treatment while minimizing additional physiological stress.

### Trauma, rescue surgery, and systems of care

Trauma continues to define the breadth of acute care surgery, demanding rapid assessment, decisive intervention, and seamless coordination across multiple specialties. The studies in this Research Topic emphasize that successful trauma management depends as much on organized systems of care and physiological prioritization as on technical operative skill.

An outstanding example is provided by Gu et al., who report survival after recurrent traumatic cardiac arrest caused by splenic rupture. Because transfer to the operating theatre would have introduced a critical delay, definitive hemorrhage control was achieved through emergency laparotomy performed directly in the trauma resuscitation area. Although exceptional, the case reinforces a fundamental principle of trauma care: when a reversible surgical cause of circulatory collapse is identified, definitive intervention must proceed without unnecessary logistical barriers.

The complexity of physiological assessment following major injury is explored by Hefny et al., whose analysis of patients with severe blunt chest trauma identified neurological impairment, mechanical ventilation, and overall physiological severity as the strongest predictors of mortality. Their findings demonstrate that thoracic injuries should rarely be considered in isolation. Instead, outcomes are determined by the cumulative burden of multisystem trauma and the quality of coordinated critical care delivered during the early post-injury period.

The concept of rescue surgery extends beyond trauma itself. Xie et al. examined secondary thoracotomy for postoperative hemorrhage following pulmonary resection, identifying extensive pleural adhesions and smoking history as important risk factors. Their observations underscore the importance of meticulous intraoperative hemostasis and targeted postoperative surveillance in patients at increased risk of bleeding. Likewise, Du et al. evaluated endoscopic treatment of gastrointestinal bleeding after cardiac surgery, demonstrating that carefully selected patients can undergo diagnostic and therapeutic endoscopy safely despite the competing challenges of anticoagulation and recent major surgery.

Reliable vascular access is another prerequisite for effective resuscitation. Gu et al. assessed ultrasound-guided axillary vein catheterization in severely injured patients and confirmed high technical success while emphasizing the need for careful attention to catheter positioning and thrombotic complications. Their experience illustrates that procedural quality extends beyond successful insertion to include appropriate verification and follow-up.

Alongside these clinical advances, digital technologies are beginning to support trauma team performance. Bruckelt et al. evaluated *TraumaFlow*, a computerized decision-support platform designed to integrate Advanced Trauma Life Support principles into real-time resuscitation. Although it did not reduce perceived workload, the system improved documentation and reduced omissions during patient management, suggesting that its principal value lies in strengthening communication, protocol adherence, and quality assurance rather than replacing clinical decision-making.

Taken together, these contributions highlight the continued evolution of trauma care from an exclusively procedure-driven discipline toward an integrated system that combines rapid diagnosis, structured resuscitation, multidisciplinary teamwork, and selective technological support. The consistent message is that innovation delivers meaningful benefit only when embedded within well-organized clinical pathways that translate timely decisions into effective patient care.

### Open abdomen, reconstruction, critical care, and complex wound management

As survival following severe trauma, abdominal sepsis, and damage-control surgery continues to improve, attention has increasingly shifted toward the management of patients beyond the initial operation. The contributions in this Research Topic emphasize that successful acute care surgery is measured not only by survival or definitive source control, but also by restoration of function, prevention of secondary complications, and the quality of multidisciplinary postoperative care.

This evolution is reflected in the work of Ioannidis et al., who describe the early clinical application of a vertical fascial traction system used in combination with negative-pressure wound therapy for the open abdomen. Their experience suggests that continuous fascial approximation may improve delayed primary closure while reducing repeated operative manipulation. More importantly, it reinforces a broader change in philosophy: temporary abdominal closure should be viewed as the beginning of a reconstructive strategy rather than the endpoint of damage-control surgery.

Understanding the physiological consequences of abdominal compartment syndrome remains equally important. Using an experimental canine model, Nalbanti et al. demonstrated progressive renal cortical hypoxia during intra-abdominal hypertension despite relatively preserved systemic hemodynamics. Although these findings require clinical validation, they suggest that regional tissue monitoring may identify evolving organ dysfunction before conventional physiological parameters deteriorate, potentially allowing earlier intervention.

The reconstructive perspective is further developed by Hou et al., who advocate a shift from simple fascial closure toward restoration of abdominal wall function. Patients surviving severe abdominal emergencies often face prolonged disability related to muscle loss, impaired abdominal mechanics, and complex incisional hernias. Their work highlights that long-term functional recovery should be considered an integral outcome of emergency surgery rather than a separate reconstructive objective.

Soft-tissue reconstruction presents similar challenges. Kim et al. demonstrate how delayed referral of trauma patients with complex wounds increases healthcare utilization and prolongs recovery, supporting closer collaboration between trauma and reconstructive services. In parallel, Ma et al. report successful management of perianal necrotizing fasciitis through repeated debridement combined with negative-pressure wound therapy, illustrating the value of coordinated surgical, wound-care, and reconstructive strategies in achieving durable healing.

Several studies also emphasize that postoperative outcomes depend heavily on critical care and nursing practice. Ping et al., through a systematic review and meta-analysis, show that guideline-based interventions can safely reduce the use of physical restraints in intensive care by combining delirium prevention, repeated assessment, staff education, and early mobilization. Likewise, Lv et al. demonstrate that bundled nursing interventions reduce pressure ulcer incidence while improving patient satisfaction and overall quality of care. Although these interventions may appear removed from the operating theatre, they directly influence recovery after major emergency surgery.

Finally, Zhang et al. explore the expanding role of specially trained surgical care nurses performing selected emergency procedures in carefully defined clinical environments. Their review suggests that competency-based training and robust clinical governance may improve access to life-saving interventions in rural, military, and disaster settings where immediate surgical expertise is unavailable, while emphasizing that such workforce expansion should complement rather than replace specialist surgical care.

Collectively, these studies broaden the scope of acute care surgery beyond emergency intervention alone. Modern ACS now encompasses reconstruction, rehabilitation, intensive care, specialized nursing, and multidisciplinary collaboration, recognizing that optimal outcomes depend on continuity of care throughout the patient's recovery rather than solely on technical success in the operating room.

### Rare emergencies, future directions, and conclusions

Although uncommon conditions account for only a small proportion of emergency surgical practice, they frequently challenge established diagnostic pathways and demonstrate the importance of flexibility in clinical decision-making. The case reports included in this Research Topic therefore contribute more than isolated observations; together, they reinforce principles that remain applicable across the wider spectrum of acute care surgery.

Pittacolo et al. describe the development of a triple gastro-colic fistula following replacement of a percutaneous endoscopic gastrostomy tube, reminding clinicians to consider visceral malposition whenever unexplained sepsis, diarrhea, or feeding intolerance develops after tube exchange. Kalathia et al. report penile strangulation caused by a metallic wedding ring, illustrating how prompt decompression, careful tissue protection, and the appropriate use of readily available instruments can prevent irreversible ischemic injury. In another unusual presentation, Lu et al. describe pneumomediastinum secondary to acute gastric dilatation after binge eating, emphasizing the need to recognize uncommon pathophysiological mechanisms when evaluating patients with acute thoracoabdominal symptoms.

Attention to anatomical detail is equally important. The correction published by Almansour et al. concerning bilateral inferior phrenic arteries arising from the coeliac trunk serves as a reminder that accurate anatomical description remains fundamental to trauma surgery, upper gastrointestinal procedures, and interventional radiology. Even seemingly minor refinements to the published literature contribute directly to safer operative planning and improved patient care.

Taken together, these reports highlight one of the defining characteristics of acute care surgery: the ability to adapt established surgical principles to unexpected clinical scenarios. While advanced technologies continue to transform emergency care, success still depends on broad anatomical knowledge, sound clinical judgement, and the willingness to reconsider initial diagnostic assumptions when the clinical picture evolves.

Beyond the individual studies, several broader themes emerge from this Research Topic. First, emergency surgical care is becoming increasingly individualized. Prediction models, advanced imaging, minimally invasive techniques, therapeutic endoscopy, and structured perioperative pathways all contribute to more precise decision-making, allowing treatment to be tailored to patient physiology rather than determined solely by disease classification.

Second, innovation is progressively extending beyond the operating room. Enhanced recovery protocols, antimicrobial stewardship, multidisciplinary critical care, abdominal wall reconstruction, specialized nursing practice, and digital clinical support all influence outcomes that matter to patients, including recovery, functional independence, and quality of life. The consistent message throughout the collection is that technical success alone is no longer sufficient; optimal results depend on coordinated care delivered across the entire patient journey.

The Research Topic also highlights priorities for future investigation. Many of the advances described require prospective validation through multicentre collaboration, standardized outcome reporting, and rigorous assessment across diverse healthcare systems. Emerging technologies—including artificial intelligence, decision-support platforms, and advanced physiological monitoring—should complement clinical expertise while remaining transparent, externally validated, and integrated into routine surgical workflows.

Finally, the diversity of methodologies represented across the collection deserves recognition. Systematic reviews synthesize existing evidence, observational studies identify clinically relevant risk factors, experimental investigations improve understanding of disease mechanisms, implementation research evaluates healthcare delivery, and carefully documented case reports broaden awareness of uncommon but important surgical problems. Together, these complementary approaches provide a balanced overview of the current state of acute care surgery while identifying opportunities for future progress.

Acute care surgery has always evolved in response to changing clinical needs. The studies assembled in this Research Topic demonstrate that its future will be defined not by any single technological innovation but by the successful integration of evidence-based practice, multidisciplinary collaboration, and patient-centered care. Timely diagnosis, effective source control, physiological optimization, technical excellence, and continuous clinical reassessment remain the foundations of the specialty. The challenge ahead is to strengthen these principles through innovation while ensuring that advances remain accessible, rigorously evaluated, and focused on improving outcomes for patients requiring emergency surgical care.

